# Supplementary material for: From homogeneous to heterogeneous network alignment via colored graphlets
Source: Sci Rep. 2018 Aug 21;8:12524. doi: 10.1038/s41598-018-30831-w (PMC6104050; doi:10.1038/s41598-018-30831-w)
Supplement: Supplementary file 1 — Supplementary information [file 41598_2018_30831_MOESM1_ESM.pdf]

# Supplementary information for From homogeneous to heterogeneous network alignment via colored graphlets

**Shawn Gu[1], John Johnson[1], Fazle E. Faisal[1,2], and Tijana Milenković[1,2,*]**

[1] Department of Computer Science and Engineering, University of Notre Dame, Notre Dame, IN, 46556, USA

[2] Eck Institute for Global Health and Interdisciplinary Center for Network Science and Applications (iCeNSA), University of Notre Dame, Notre Dame, IN, 46556, USA

[*] To whom correspondence should be addressed (email: tmilenko@nd.edu)

Here, we include all results.

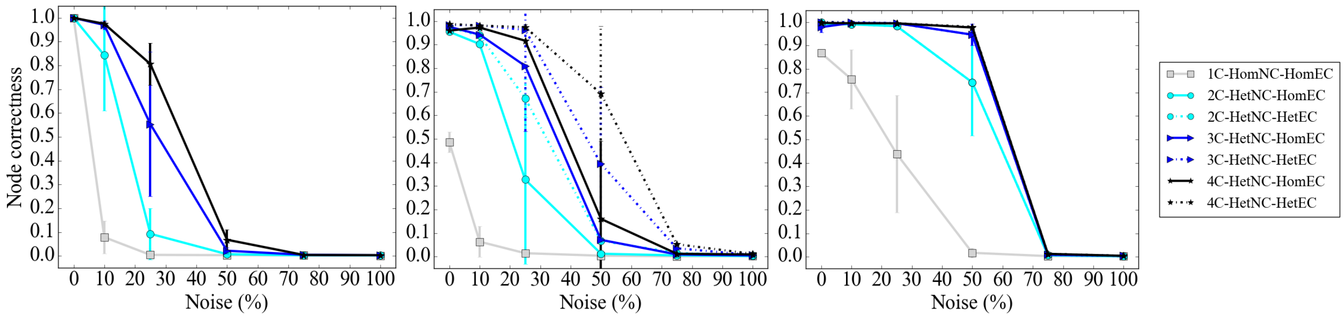


**(a)** **(b)** **(c)**

**Supplementary Figure S1.** Detailed alignment quality results regarding the effect of the **number of node colors** on alignment quality as a function of noise level for **synthetic, specifically geometric**, networks using (a) WAVE, (b) MAGNA++, and (c) SANA. Gray squares, light blue circles, dark blue triangles, and black stars indicate the aligned networks containing one, two, three, and four node colors, respectively. For two or more node colors, solid lines represent using HetNC-HomEC, and dashed lines represent using HetNC-HetEC.

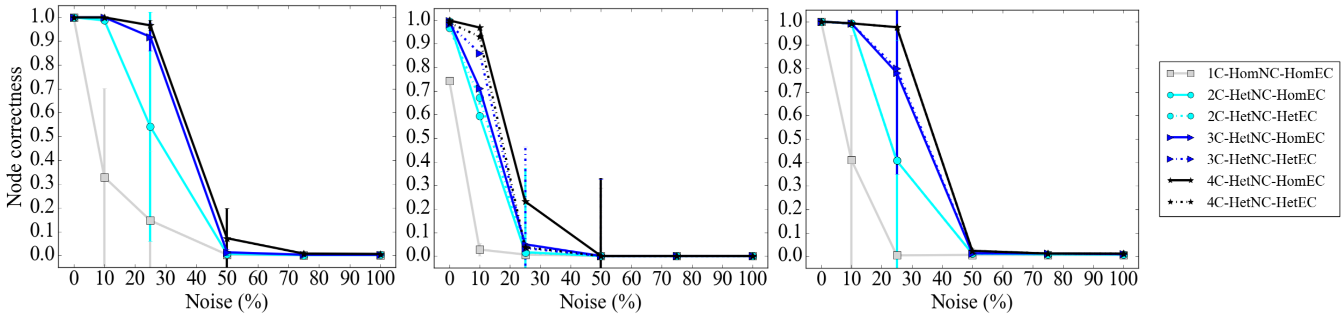


**(a)** **(b)** **(c)**

**Supplementary Figure S2.** Detailed alignment quality results regarding the effect of the **number of node colors** on alignment quality as a function of noise level for **synthetic, specifically scale-free**, networks using (a) WAVE, (b) MAGNA++, and (c) SANA. The figure can be interpreted in the same way as Supplementary Figure S1.

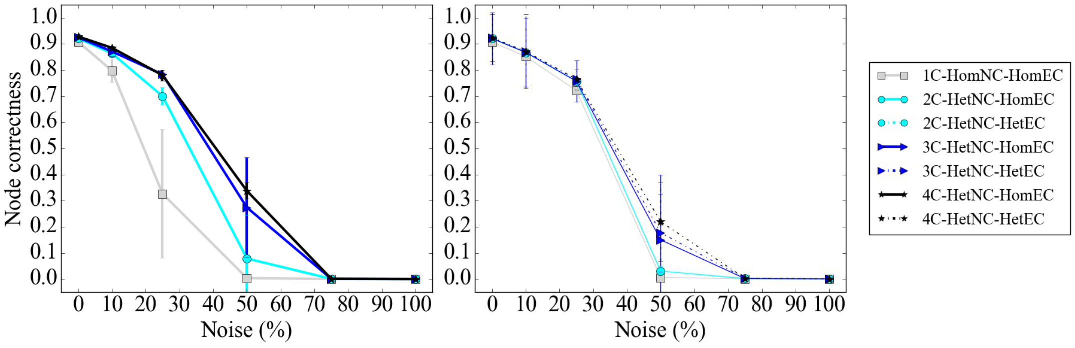


**(a)** **(b)**

**Supplementary Figure S3.** Detailed alignment quality results regarding the effect of the **number of node colors** on alignment quality as a function of noise level for **PPI, specifically APMS-Expr**, networks using (a) WAVE and (b) SANA. The figure can be interpreted in the same way as Supplementary Figure S1. Recall that for these larger networks, we have not run MAGNA++ due to its high computational complexity.

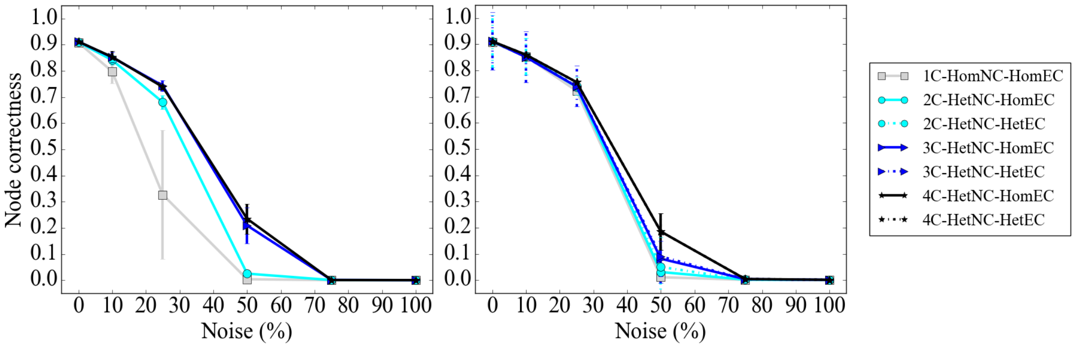


**(a)** **(b)**

**Supplementary Figure S4.** Detailed alignment quality results regarding the effect of the **number of node colors** on alignment quality as a function of noise level for **PPI, specifically APMS-Seq**, networks using (a) WAVE and (b) SANA. The figure can be interpreted in the same way as Supplementary Figure S1. Recall that for these larger networks, we have not run MAGNA++ due to its high computational complexity.

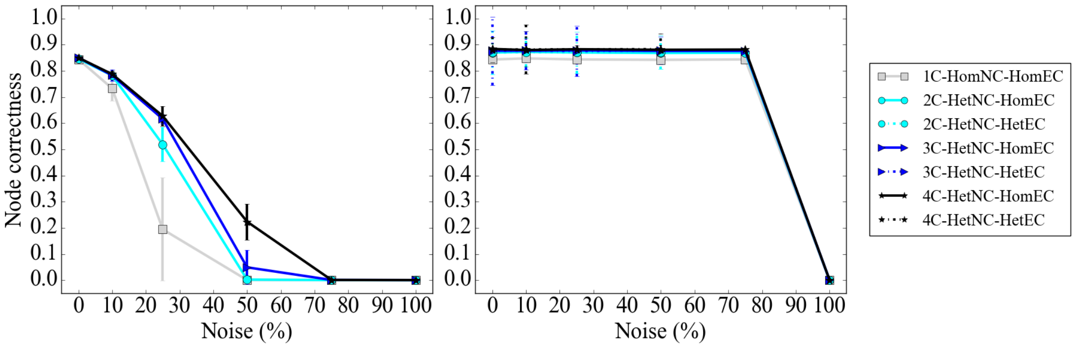


**(a)** **(b)**

**Supplementary Figure S5.** Detailed alignment quality results regarding the effect of the **number of node colors** on alignment quality as a function of noise level for **PPI, specifically Y2H-Expr**, networks using (a) WAVE and (b) SANA. The figure can be interpreted in the same way as Supplementary Figure S1. Recall that for these larger networks, we have not run MAGNA++ due to its high computational complexity.

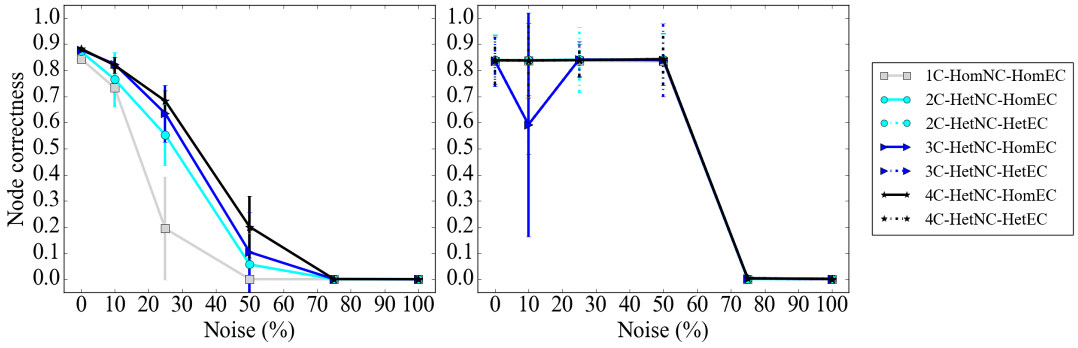


**(a)** **(b)**

**Supplementary Figure S6.** Detailed alignment quality results regarding the effect of the **number of node colors** on alignment quality as a function of noise level for **PPI, specifically Y2H-Seq**, networks using (a) WAVE and (b) SANA. The figure can be interpreted in the same way as Supplementary Figure S1. Recall that for these larger networks, we have not run MAGNA++ due to its high computational complexity.

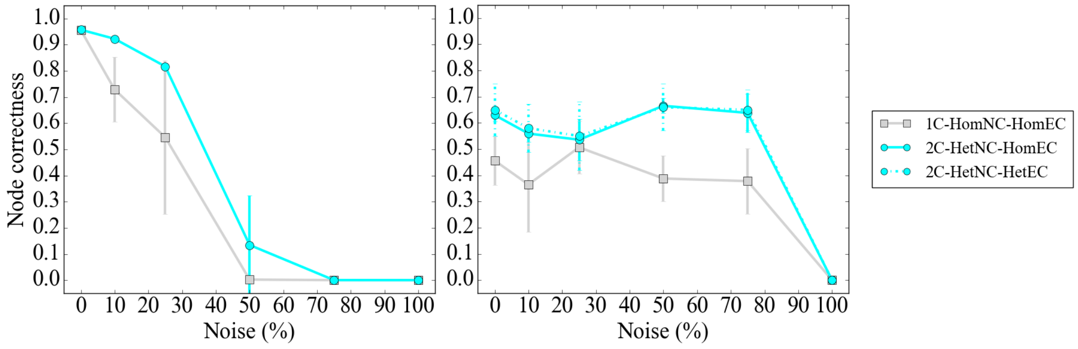


**(a)** **(b)**

**Supplementary Figure S7.** Detailed alignment quality results regarding the effect of the **number of node colors** on alignment quality as a function of noise level for **protein-GO, specifically protein-GO-APMS**, networks using (a) WAVE and (b) SANA. The figure can be interpreted in the same way as Supplementary Figure S1. Recall that for these larger networks, we have not run MAGNA++ due to its high computational complexity.

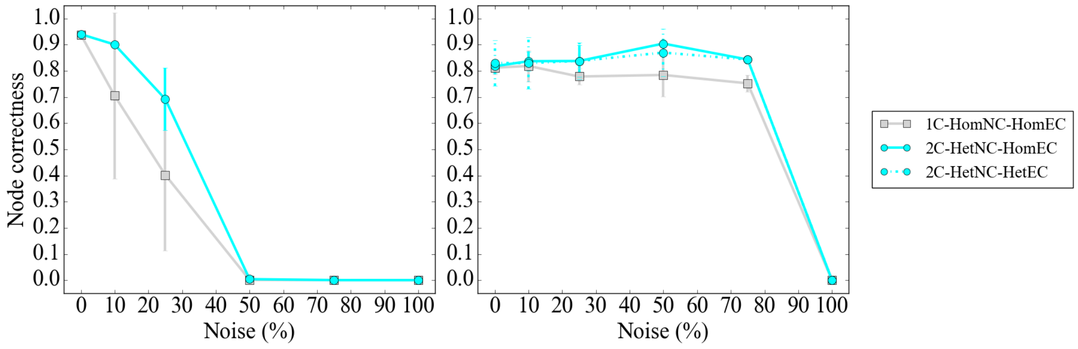


**(a)** **(b)**

**Supplementary Figure S8.** Detailed alignment quality results regarding the effect of the **number of node colors** on alignment quality as a function of noise level for **protein-GO, specifically protein-GO-Y2H**, networks using (a) WAVE and (b) SANA. The figure can be interpreted in the same way as Supplementary Figure S1. Recall that for these larger networks, we have not run MAGNA++ due to its high computational complexity.

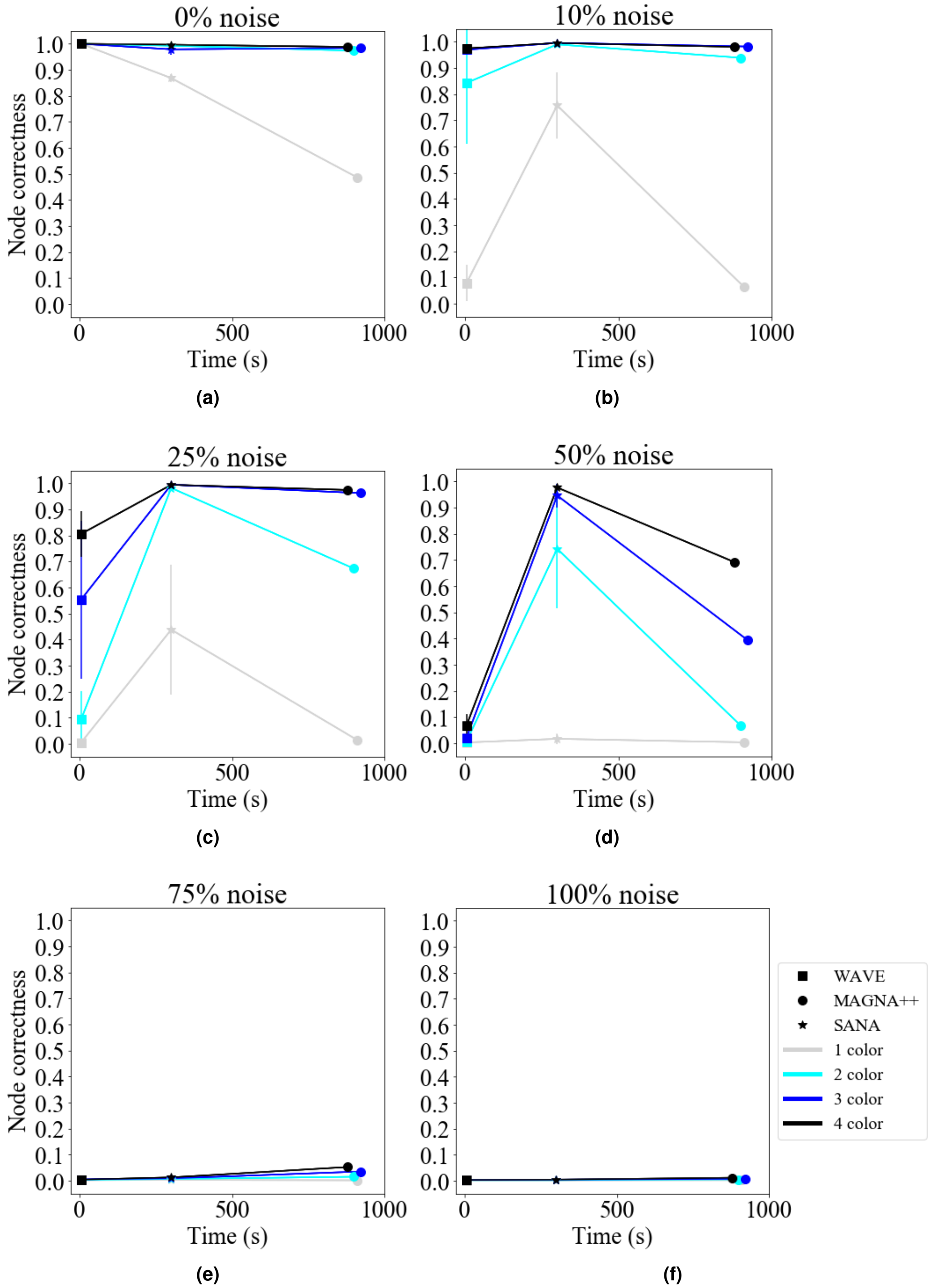


**Supplementary Figure S9.** Detailed results comparing the **running time** and effect of the **number of node colors** for different methods for all tested noise levels on **synthetic, specifically geometric**, networks. The *x*-axis the the running time of the method, and the *y*-axis is the alignment quality. Here we use different shapes to represent the different methods and different colored lines to represent how many node colors are used. Lines are drawn between methods using the same number of colors.

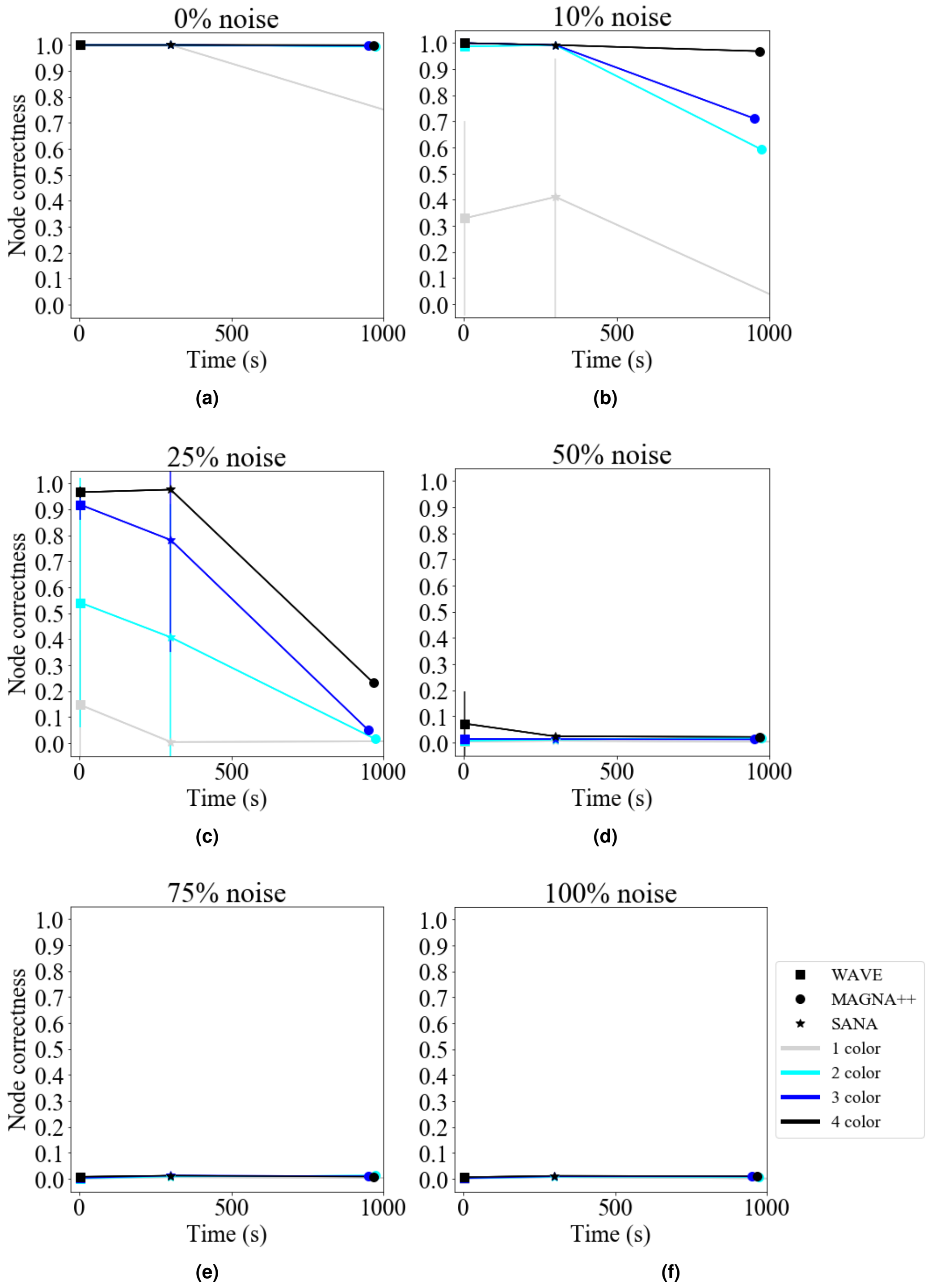


**Supplementary Figure S10.** Detailed results comparing the **running time** and effect of the **number of node colors** for different methods for all tested noise levels on **synthetic, specifically scale-free**, networks. The figure can be interpreted in the same way as Supplementary Figure S9.

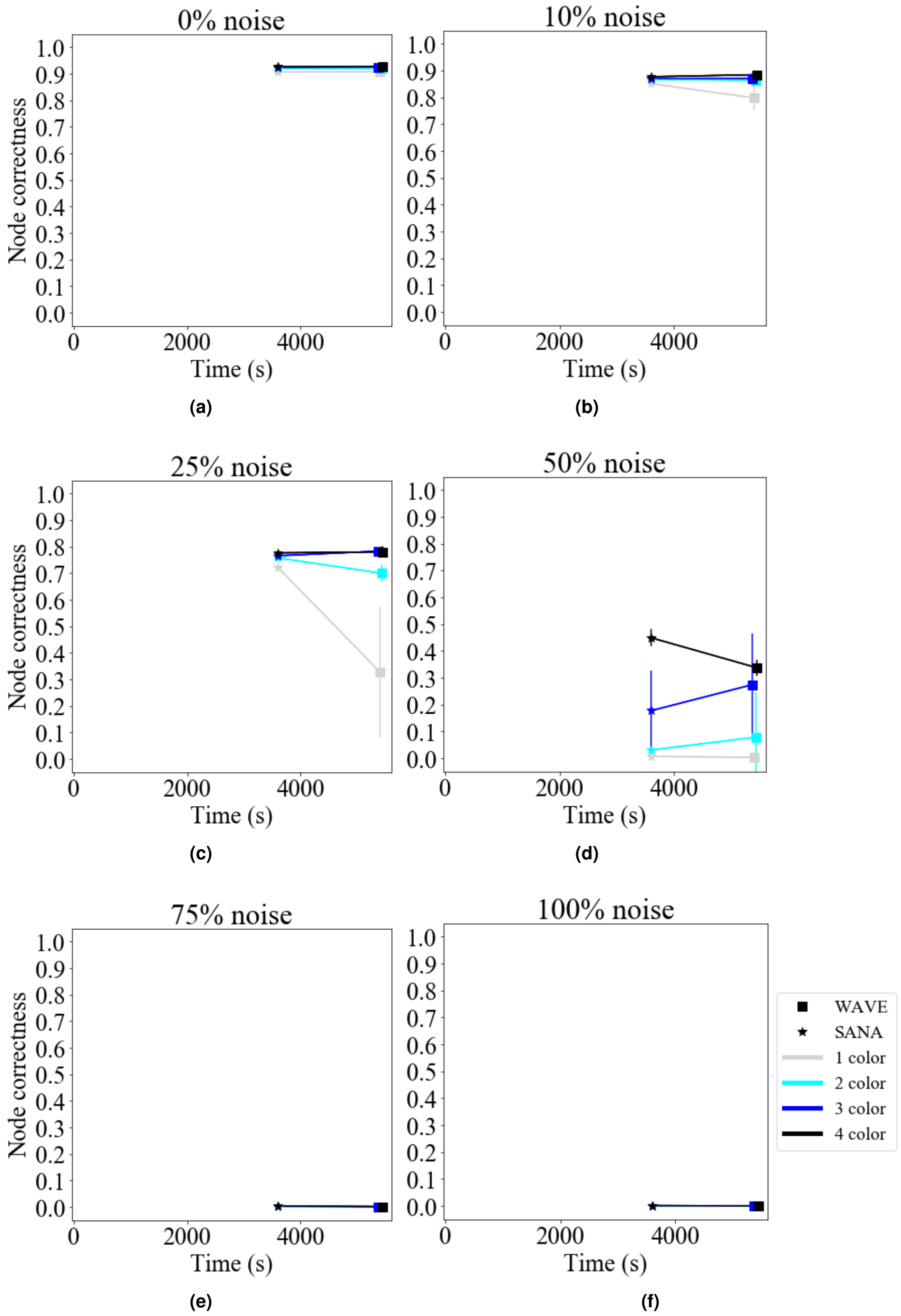


**Supplementary Figure S11.** Detailed results comparing the **running time** and effect of the **number of node colors** for different methods for all tested noise levels on **PPI, specifically APMS-Expr**, networks. The figure can be interpreted in the same way as Supplementary Figure S9.

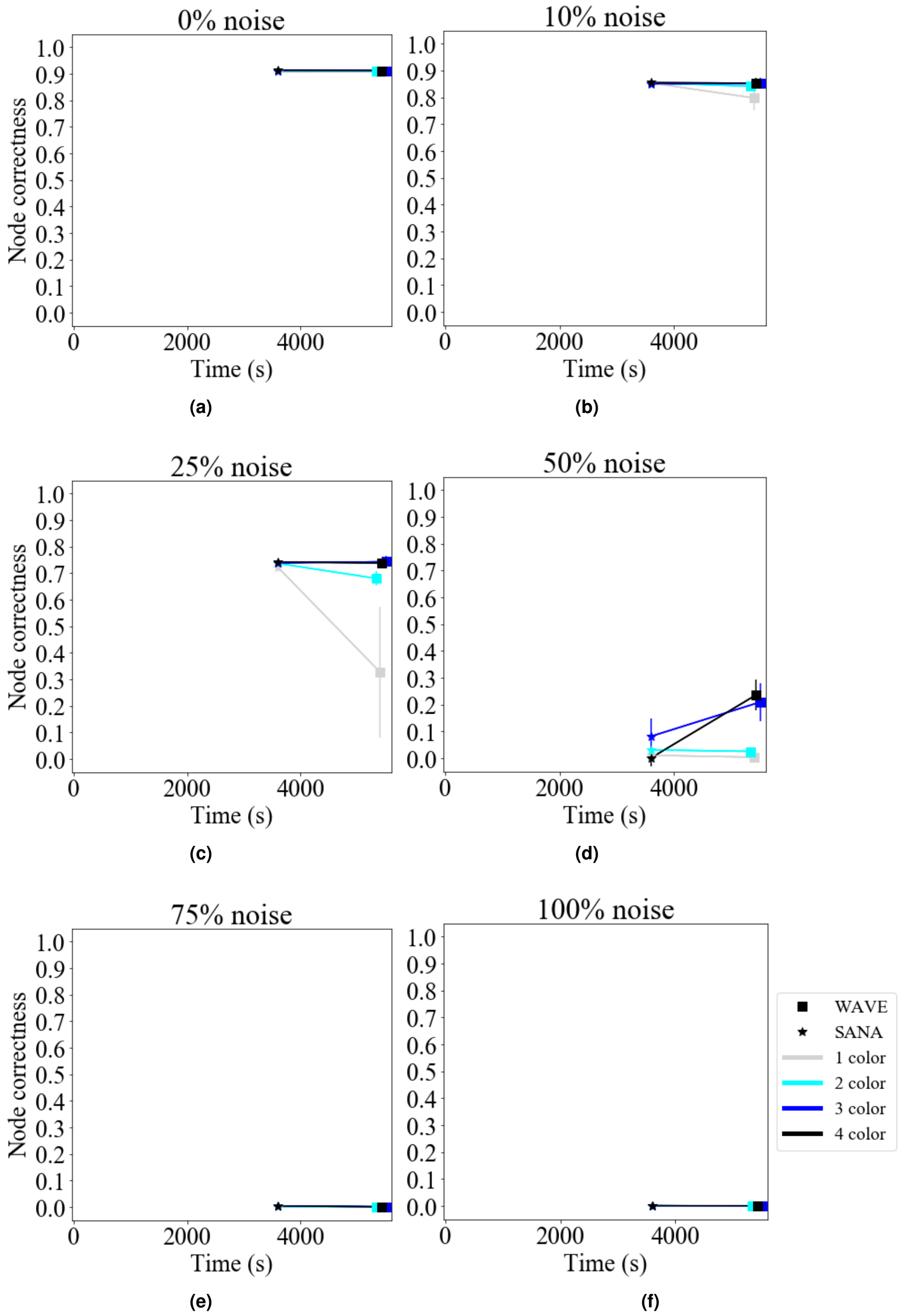


**Supplementary Figure S12.** Detailed results comparing the **running time** and effect of the **number of node colors** for different methods for all tested noise levels on **PPI, specifically APMS-Seq**, networks. The figure can be interpreted in the same way as Supplementary Figure S9.

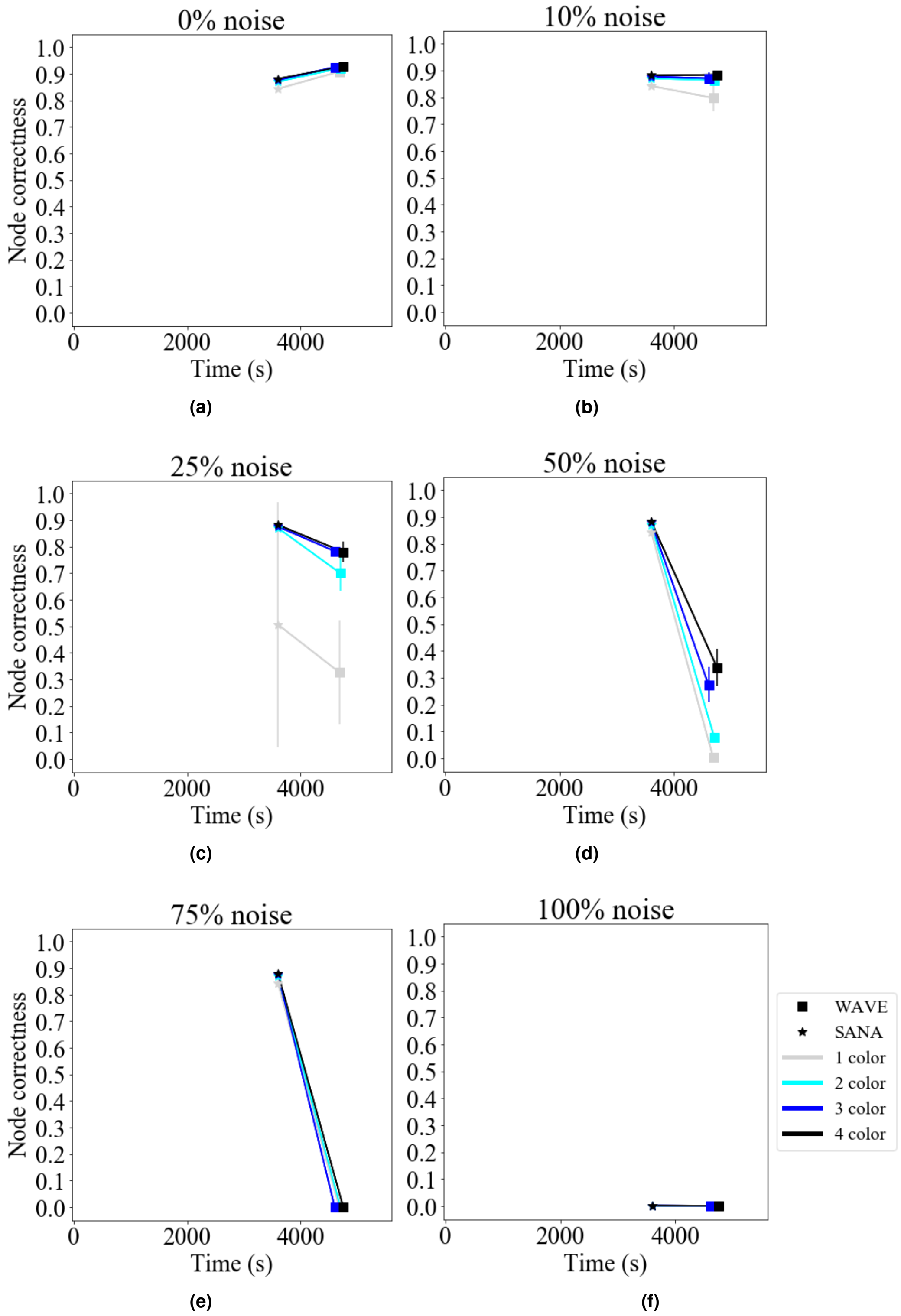


**Supplementary Figure S13.** Detailed results comparing the **running time** and effect of the **number of node colors** for different methods for all tested noise levels on **PPI, specifically Y2H-Expr**, networks. The figure can be interpreted in the same way as Supplementary Figure S9.

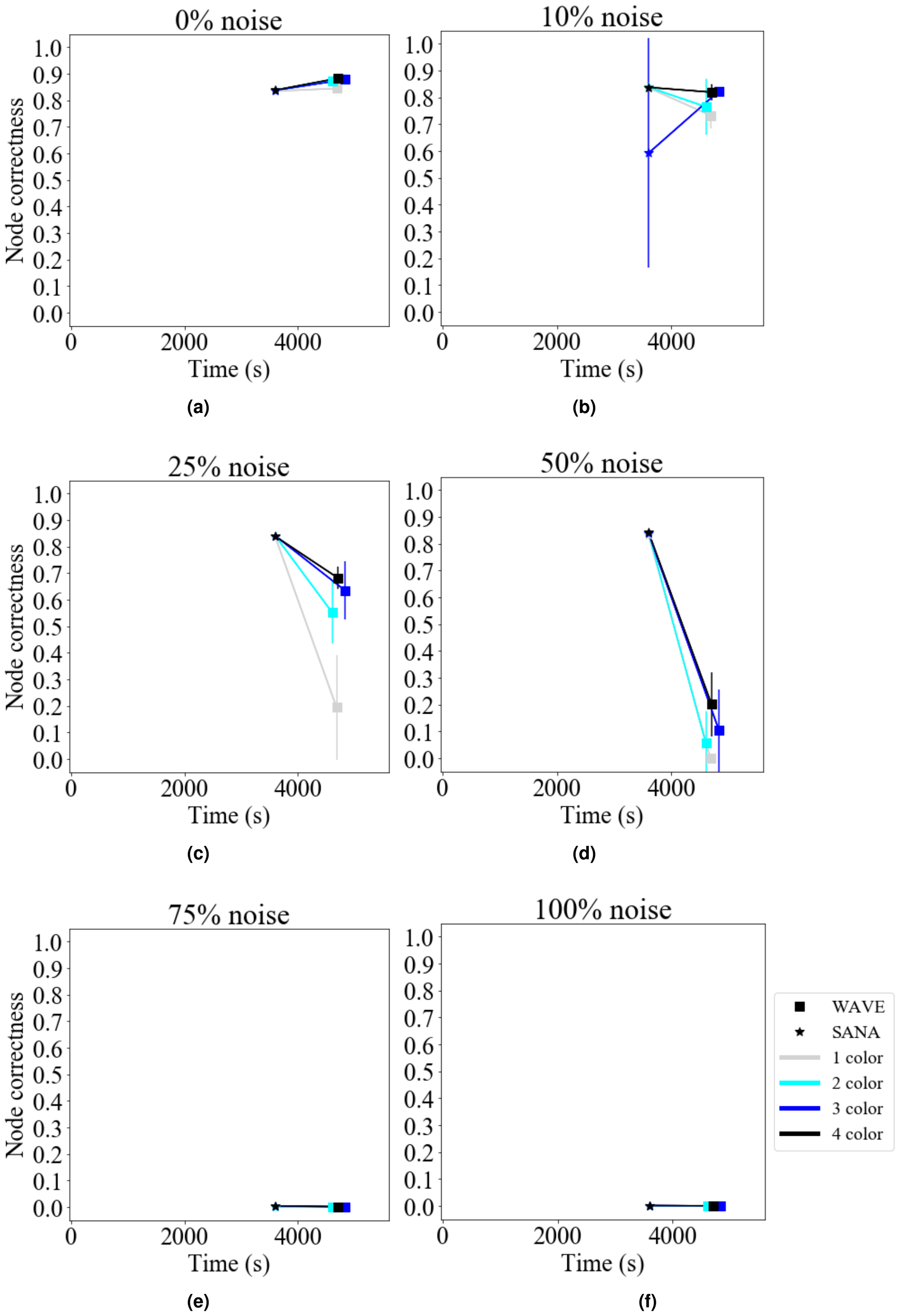


**Supplementary Figure S14.** Detailed results comparing the **running time** and effect of the **number of node colors** for different methods for all tested noise levels on **PPI, specifically Y2H-Seq**, networks. The figure can be interpreted in the same way as Supplementary Figure S9.

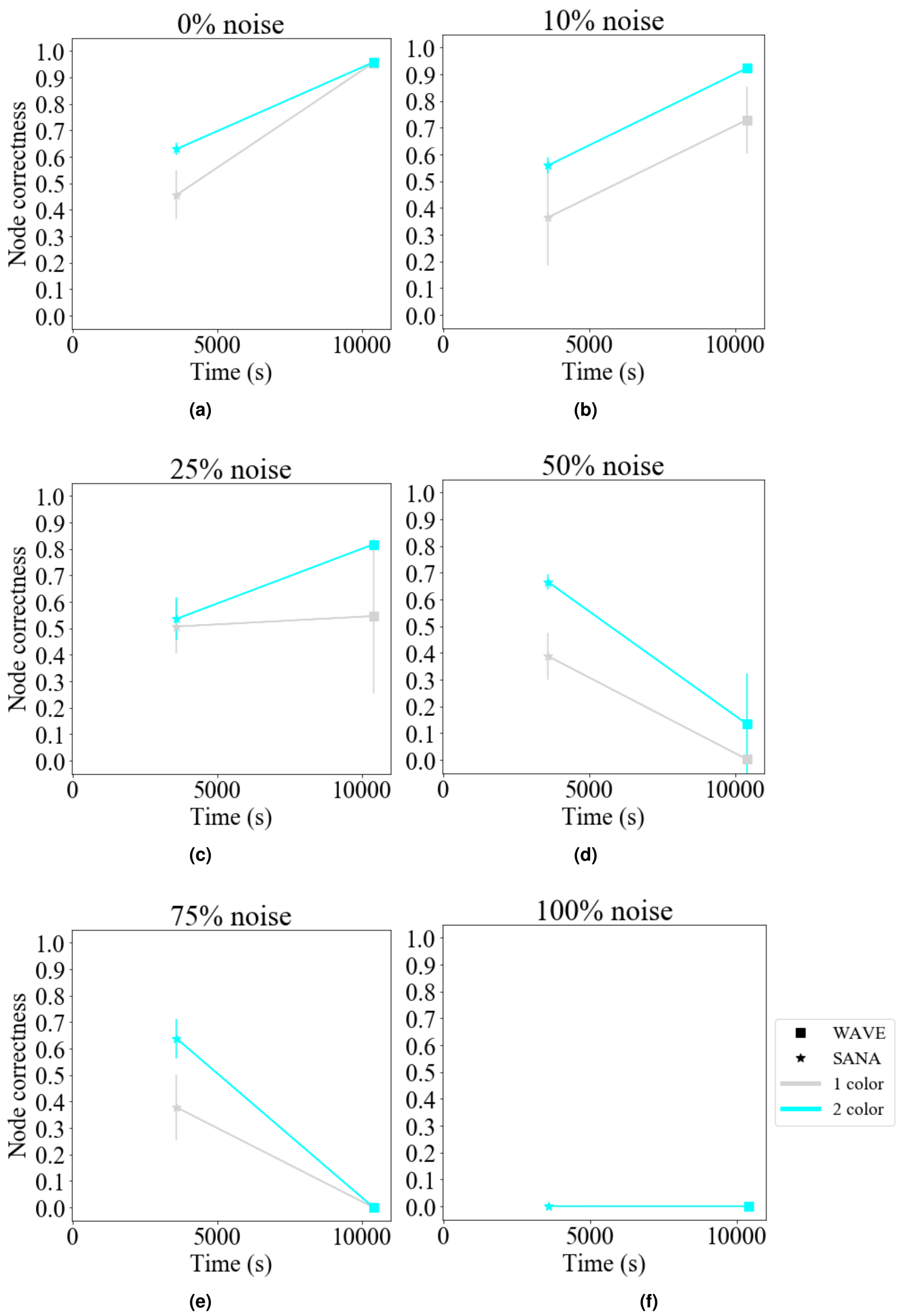


**Supplementary Figure S15.** Detailed results comparing the **running time** and effect of the **number of node colors** for different methods for all tested noise levels on **protein-GO, specifically protein-GO-APMS**, networks. The figure can be interpreted in the same way as Supplementary Figure S9.

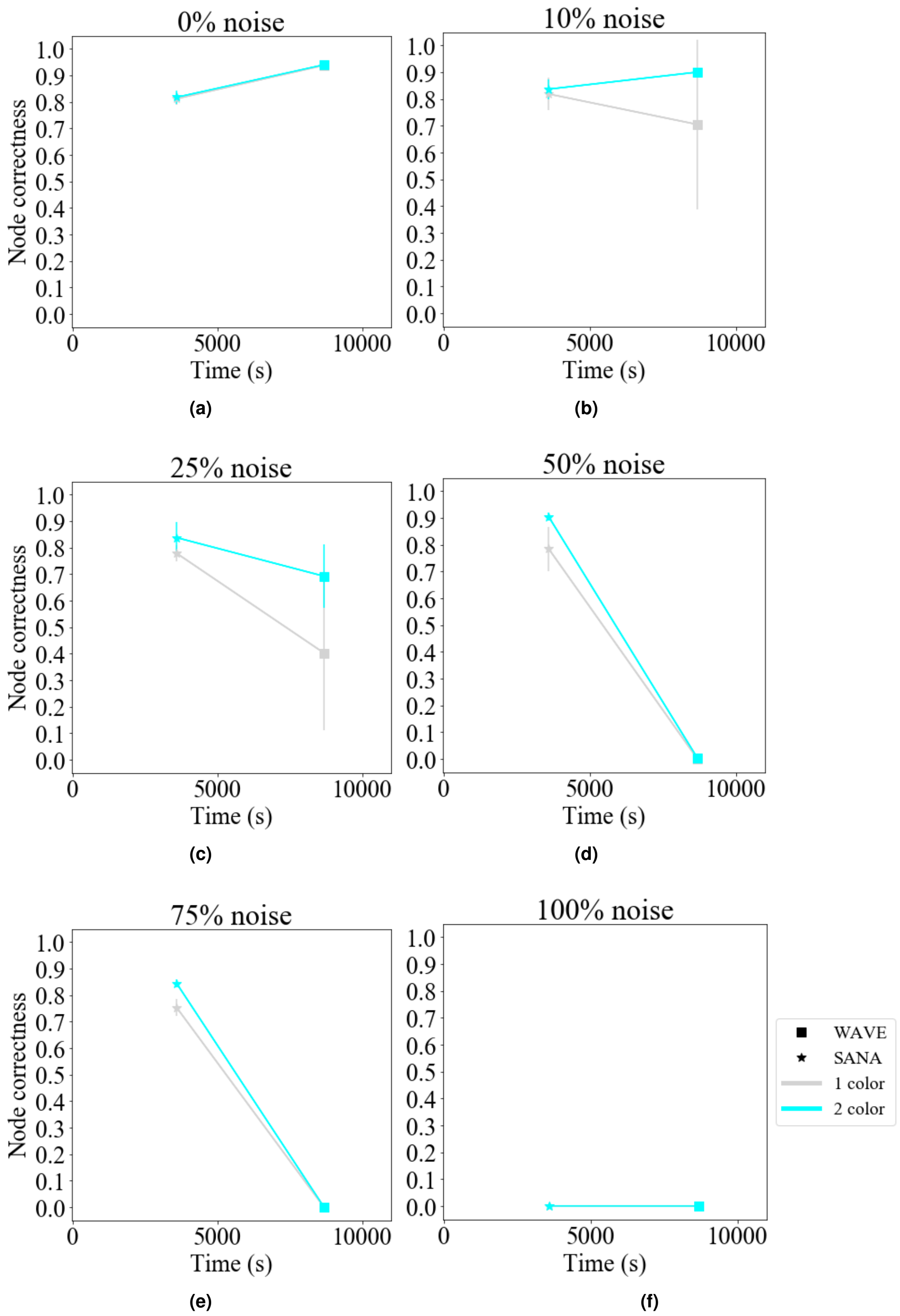


**Supplementary Figure S16.** Detailed results comparing the **running time** and effect of the **number of node colors** for different methods for all tested noise levels on **protein-GO, specifically protein-GO-Y2H**, networks. The figure can be interpreted in the same way as Supplementary Figure S9.

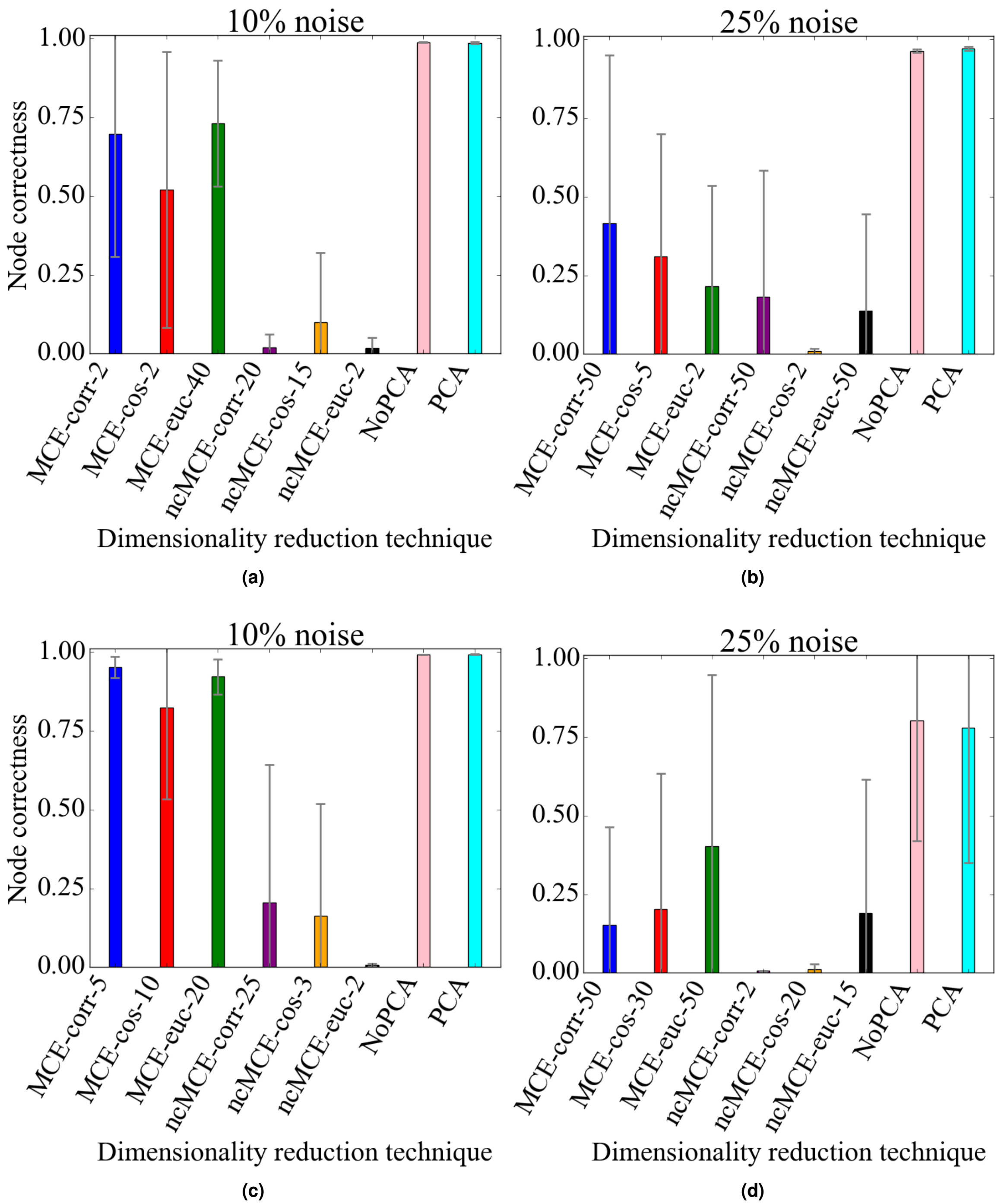


**Supplementary Figure S17.** Detailed alignment quality results regarding the effect of the **dimensionality reduction technique** on alignment quality for (a)-(b) geometric and (c)-(d) scale-free networks using **Pearson correlation** under **WAVE**. For MCE/ncMCE, we indicate the number of dimensions out of those tested (2, 3, 5, 10, 15, 20, 25, 30, 40, 50) that gives the best results, i.e., the highest alignment quality. NoPCA corresponds to using no dimensionality reduction.

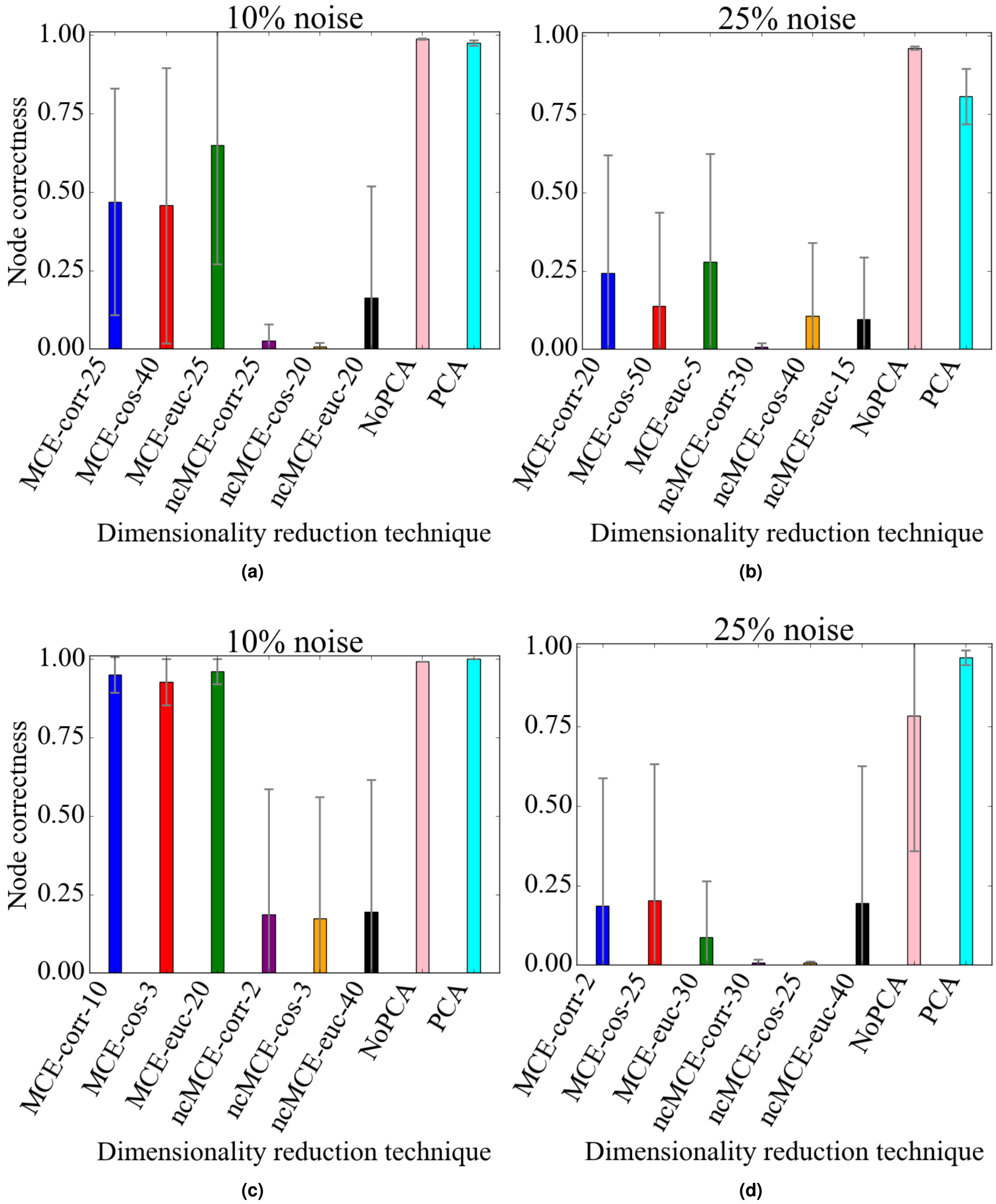


**Supplementary Figure S18.** Detailed alignment quality results regarding the effect of the **dimensionality reduction technique** on alignment quality for (a)-(b) geometric and (c)-(d) scale-free networks using **cosine similarity** under **WAVE**. For MCE/ncMCE, we indicate the number of dimensions out of those tested that gives the best results, i.e., the highest alignment quality. NoPCA corresponds to using no dimensionality reduction.

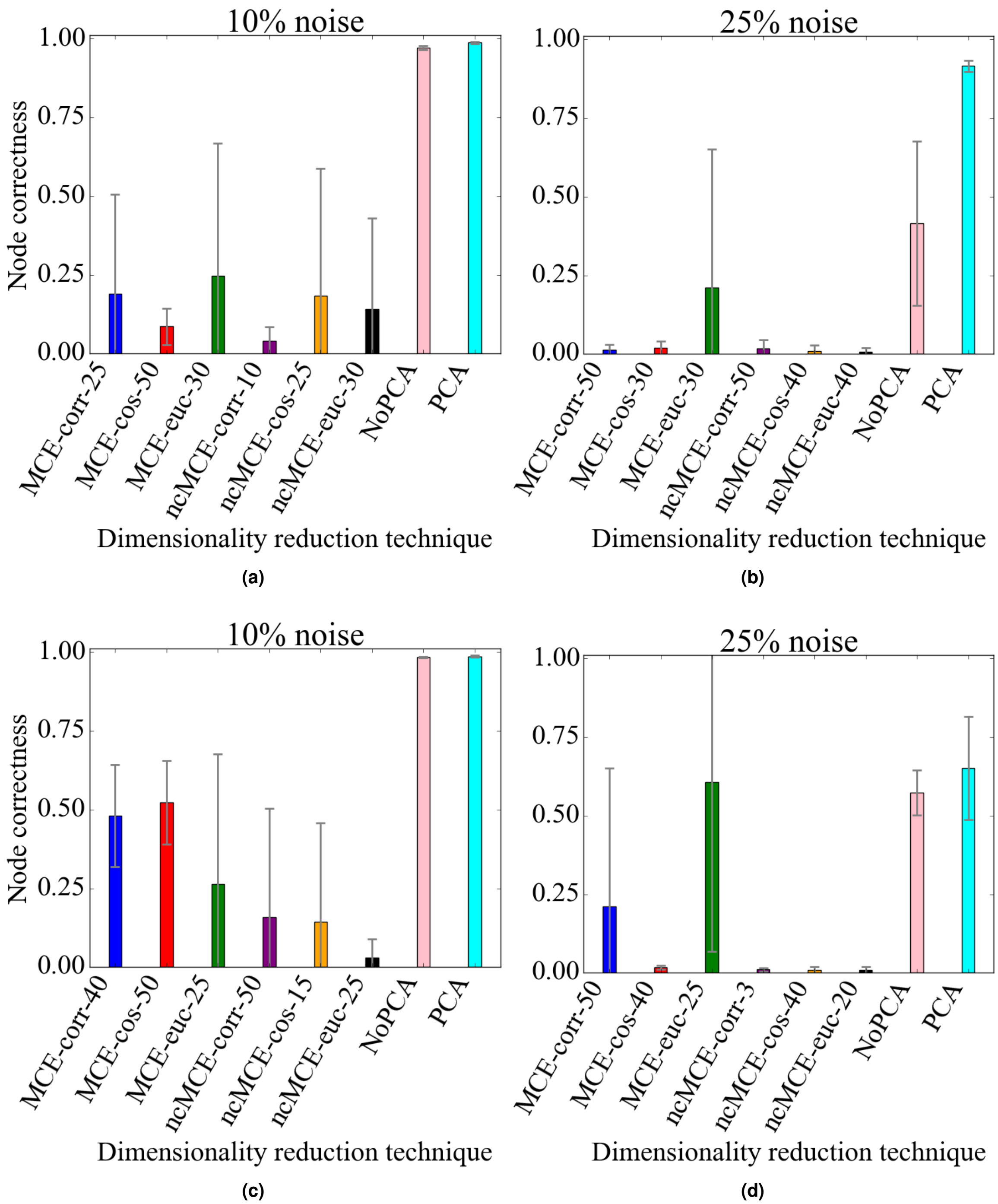


**Supplementary Figure S19.** Detailed alignment quality results regarding the effect of the **dimensionality reduction technique** on alignment quality for (a)-(b) geometric and (c)-(d) scale-free networks using the **inverse of Euclidean distance** under **WAVE**. For MCE/ncMCE, we indicate the number of dimensions out of those tested that gives the best results, i.e., the highest alignment quality. NoPCA corresponds to using no dimensionality reduction.

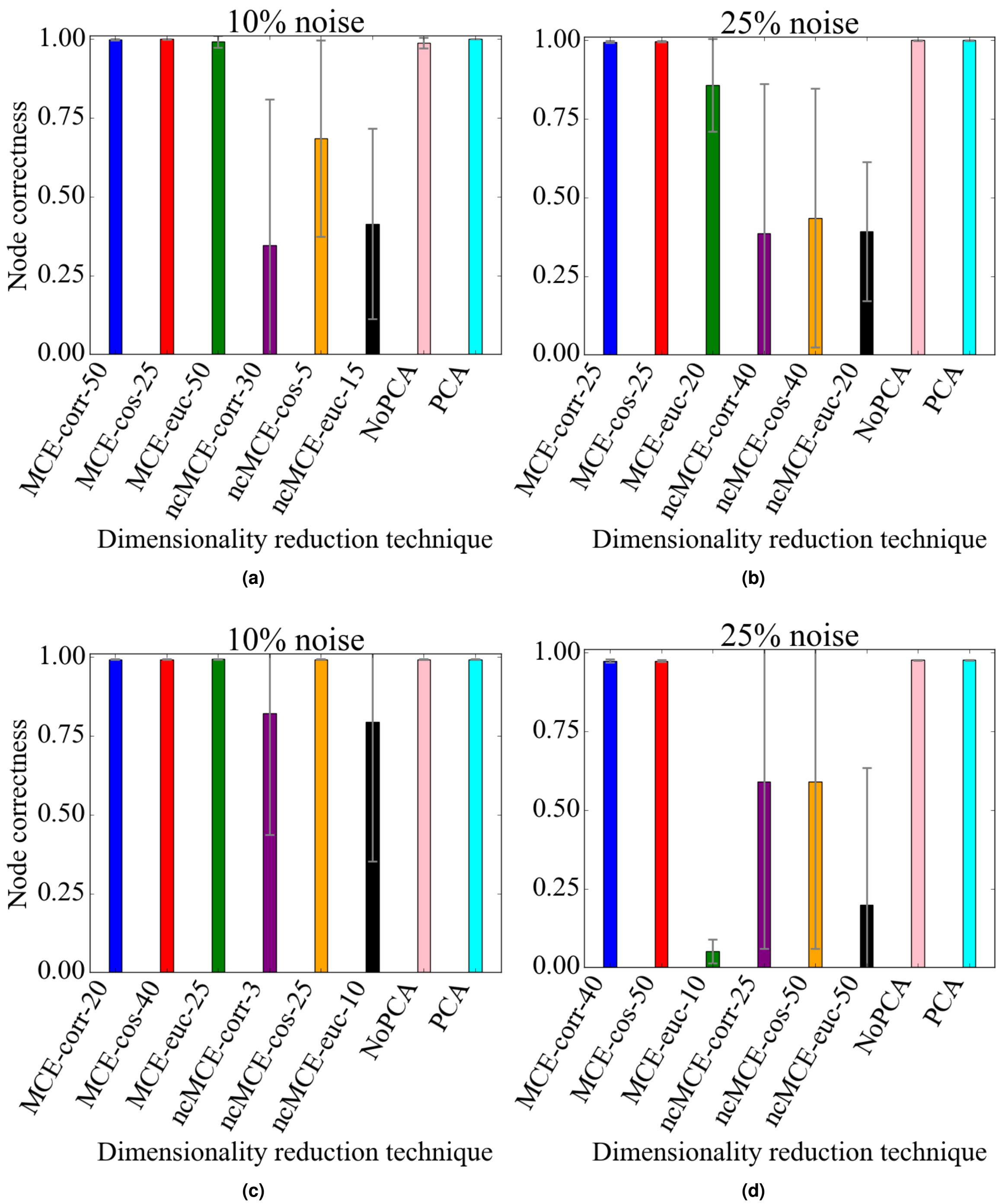


**Supplementary Figure S20.** Detailed alignment quality results regarding the effect of the **dimensionality reduction technique** on alignment quality for (a)-(b) geometric and (c)-(d) scale-free networks using **Pearson correlation** under **SANA**. For MCE/ncMCE, we indicate the number of dimensions out of those tested that gives the best results, i.e., the highest alignment quality. NoPCA corresponds to using no dimensionality reduction.

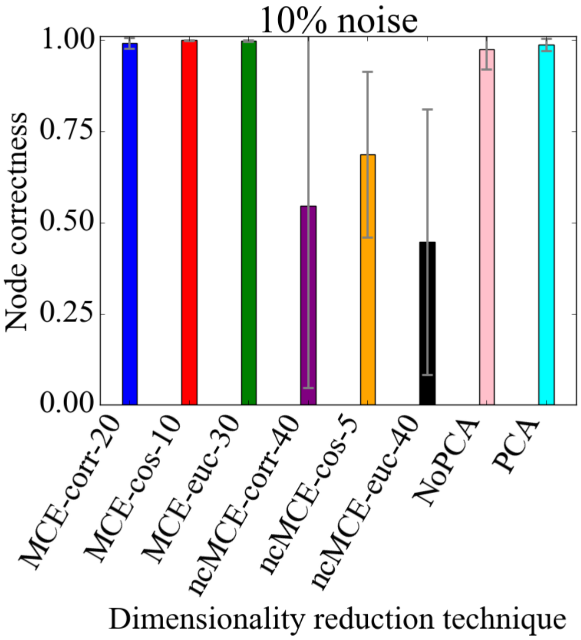


(a)

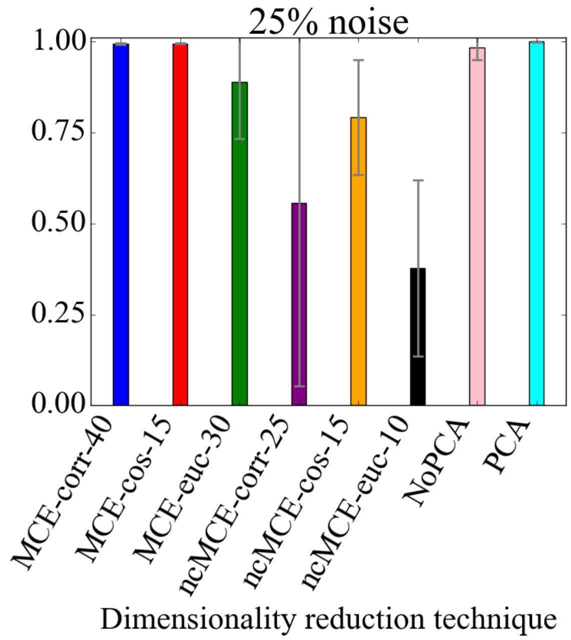


(b)

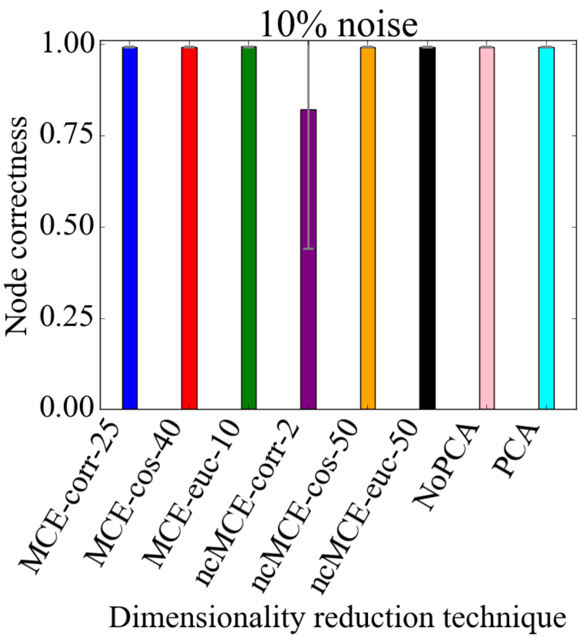


(c)

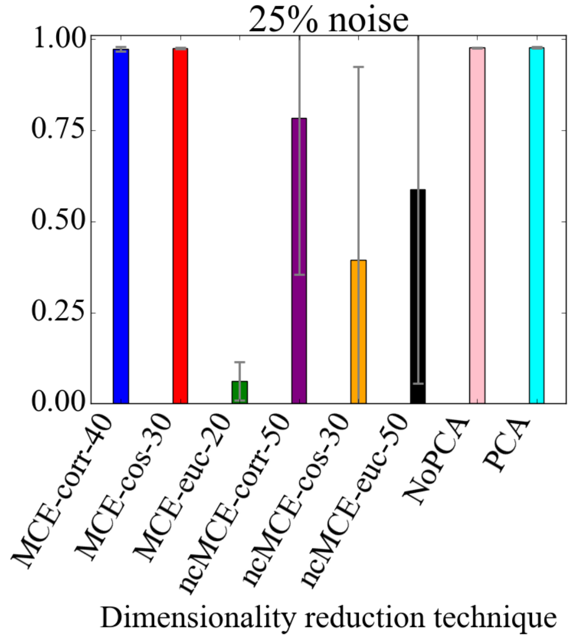


(d)

**Supplementary Figure S21.** Detailed alignment quality results regarding the effect of the **dimensionality reduction technique** on alignment quality for (a)-(b) geometric and (c)-(d) scale-free networks using **cosine similarity** under **SANA**. For MCE/ncMCE, we indicate the number of dimensions out of those tested that gives the best results, i.e., the highest alignment quality. NoPCA corresponds to using no dimensionality reduction.

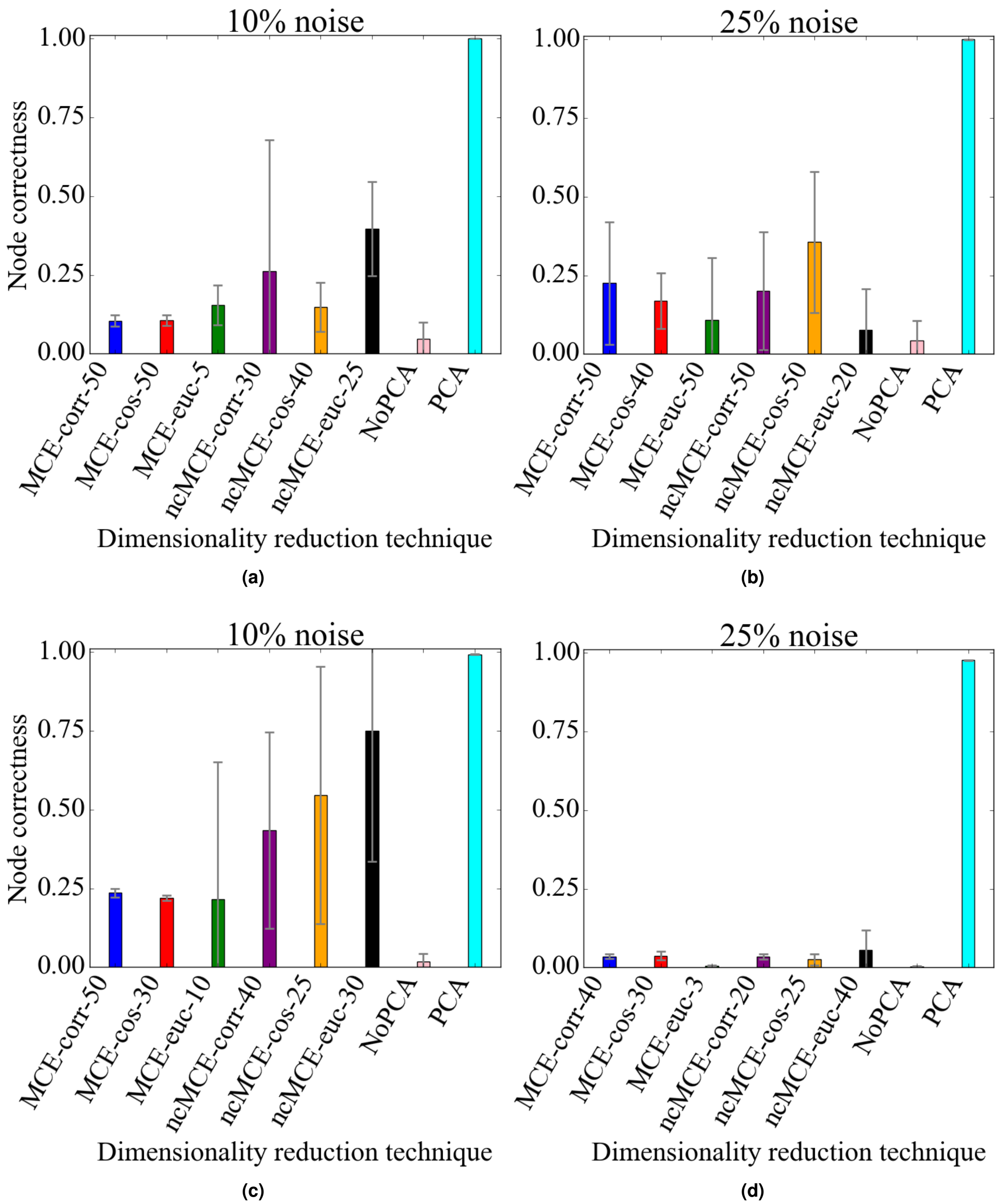


**Supplementary Figure S22.** Detailed alignment quality results regarding the effect of the **dimensionality reduction technique** on alignment quality for (a)-(b) geometric and (c)-(d) scale-free networks using the **inverse of Euclidean distance** under **SANA**. For MCE/ncMCE, we indicate the number of dimensions out of those tested that gives the best results, i.e., the highest alignment quality. NoPCA corresponds to using no dimensionality reduction.
